# Recurrent Horner's syndrome following epidural analgesia for labor: A case report

**DOI:** 10.1097/MD.0000000000018862

**Published:** 2020-01-31

**Authors:** Caroline Turbelin, Jihad Mallat

**Affiliations:** a Department of Anesthesiology and Critical Care Medicine, CH Lens, Lens, France; b Department of Critical Care Medicine, Critical Care Institute, Cleveland Clinic Abu Dhabi, Abu Dhabi, UAE.

**Keywords:** epidural analgesia, Horner's syndrome, subdural block

## Abstract

**Introduction:**

Horner's syndrome is an unusual side effect of epidural analgesia. We report an unusual symptom after epineural axis analgesia for labor, which happened three times in the same patient. Horner's syndrome occurrence following epidural catheterization should lead the anesthetic team to search for a subdural position of the catheter because of its potentially threatening complications.

**Patient's concern:**

Our patient, a 38 years old pregnant woman, was managed by the anesthetic team for the analgesia of her second labor. Anesthetic consultation pointed out that she had a history of Horner's syndrome after epidural analgesia attempt during her first pregnancy.

During our anesthetic management of her second labor, she presented, on the left side of the body, with the same symptom as she had during her first labor a few years ago, associated with high unilateral sensory block after epidural catheterization. After the total regression of neurological signs, a second catheterization attempt was followed by a contralateral isolated Horner's syndrome with no sensory block.

**Diagnosis:**

A few minutes after the induction of analgesia, our patient presented left ptosis, meiosis, and enophthalmos associated with a high homolateral sensory block. The subdural catheter position was suspected, and the catheter was removed.

**Intervention:**

Because of this repeated unusual complication and because we would not have used the catheter for an emergency C section, we chose to remove it definitively.

**Outcome:**

Our patient had a total regression of the symptoms 1 h after the catheter withdrawal. Delivery was rapid, with no complications, and she was discharged from the hospital 3 days after.

**Conclusion:**

Our observations suggest the possibility of a potential anatomical particularity of the dural canal in this patient and question the safety of performing an epidural catheterization for further procedures.

## 1 Introduction

Horner's syndrome is an unusual side effect of epidural analgesia.^[1,2]^ Its incidence is reported to be 0.5% of epidural analgesia for labor, rate increasing to 4% if top-ups are used for the realization of a cesarean section.^[3,4]^ A recent review of the literature identified 78 cases after a single attempt reported in the main medical databases.^[2]^ Horner's syndrome is described as well as a sign of benign cephalic spread of local anesthetics or as a sign of subdural block.^[2]^ More often, it is considered as a benign phenomenon.^[1,2]^ The difficulty consists in recognizing the subdural catheter position because of its potentially threatening complications such as dura-mater rupture.^[5,6]^ No clear guidelines exist about catheter management in the case of Horner's syndrome following epineural analgesia. For some authors, this isolated symptom should not lead to discontinuation of the neuraxial blockade.^[1,2]^ For others, this rare symptom could be linked to the subdural position of the catheter and then should lead to epidural analgesia disruption.^[6]^

We report this unusual complication of epineural axis analgesia for labor happening three times in the same patient at different periods of her medical history.

## 2 Case

A 32-year-old parturient, Gravida 2, Para 1, came at 38 weeks of gestation for spontaneous labor. She had a history of obesity with a body mass index of 35.3 kg/m^2^, tympanoplasty, and gastric band surgery. During her first delivery, epidural analgesia was complicated with Horner's syndrome. Rapid dilatation did not allow the anesthetic team to replace the epidural catheter then, and the patient had spontaneous vaginal birth without any analgesia.

Anesthetic consultation during her second pregnancy pointed out this complication.

For her second labor, an epidural catheter was placed without difficulty. Test dose (3 mL of 1% non-epinephrine lidocaine) was administered, and the aspiration test was negative. Twenty minutes after the first anesthetic bolus (50 mg of 1% non-epinephrine lidocaine), the pain was relieved, but the patient presented a left Horner's syndrome associated with a full and exclusive left sensory block (T4 level) without a motor blockade. Hemodynamic parameters remained stable during the whole process. The subdural catheter position was suspected, and the second placement of the epidural catheter was performed after a total regression of neurological signs and under cardiac monitoring. After a new test dose and negative aspiration, careful induction of epineural analgesia was performed, with 50 mg of non-epinephrine lidocaine divided into two injections. Fifteen minutes later, the patient presented a contralateral right Horner's syndrome associated with no sensory block. Again, hemodynamic parameters were perfectly stable during the whole procedure and surveillance. Due to the potential risks and the impossibility of using it in case of an emergency C section, the catheter was removed and not inserted again after the full disclosure of information to the patient who agreed with the anesthetic management. Once again, she had rapid labor and had no instrumental vaginal delivery with no analgesia 30 min later. Follow up did not reveal any complication, and she was discharged from the hospital 3 days later.

## 3 Discussion

Horner's syndrome, after epidural analgesia, is the result of the stellar ganglion blockade, suggesting a high level (C8–T4) of anesthetic effects.

Two hypotheses could explain the mechanisms of the repeated Horner's syndrome observed in our patient. First, the catheter was in the epidural space, but the patient might have a narrow epidural space, which adds to the pregnancy condition and high BMI could have caused an extensive cephalic spread of local anesthetics and Horner's syndrome recurrence after different epidural catheterization attempts.^[1]^ However, the clinical presentation with the unilateral sensitive and sympathetic blockade after a low volume injection does not fit this hypothesis. More often, it is observed when high volumes of local anesthetics are injected, especially in shorter women, and usually results in a bilateral sensory blockade with a unilateral higher level and Horner's syndrome due to a capillary ascension of the local anesthetic toward meningeal membrane.^[1,3]^ Horner's syndrome could be one of its clinical signs, but it also occurs with a benign cephalic spread of anesthetics, implicating accessory ways.^[1]^ The second explanation could be that the catheter was in the subdural space, but the dilation of the subdural space by the first attempt of the epidural analgesia might have modified the local anatomy and then have facilitated the second subdural catheterization. Review of the literature about subdural block pointed out to predisposing factors for this complication such as difficult block placement, rough handling and rotation of an epidural needle in the epidural space, previous back surgery, and recent lumbar puncture.^[6]^ Anatomical studies showed inter-individual variability. Indeed, a subdural space was found in only 1% to 13% of the population.^[7,8]^ Furthermore, some authors pointed out that once the subdural compartment is expanded, it is very challenging to place any subsequent injection into the subarachnoid space. These findings added to the unilateral high sensory, and sympathetic blockade disproportionate to the amount of local anesthetic injected supports the second hypothesis in our patient. Thus, we believe that our patient might have a regional anatomy variation explaining the repeated epidural failure, but only a radiological investigation would have definitively given the diagnosis, which was not done in this present case.

Regarding Horner's syndrome after epidural analgesia for labor, our PubMed search matched 24 results. A recent systematic review of the literature conducting a wider search in all the main medical databases found 78 cases reported. This review mentions 4 cases in which patients presented a recurrent Horner's syndrome and 3 cases of bilateral Horner's syndrome.^[2]^ Our PubMed search with the terms “recurrent Horner's syndrome and epidural analgesia,” only found 2 cases. One of them is particularly relevant as it describes the case of a patient in which multiple attempts of epidural analgesia for labor led to Horner's syndrome repeated occurrence.^[9]^ The research we conducted in the PubMed database for “bilateral Horner's syndrome and epidural analgesia” did not match any result.

Incidence of this adverse event is reported to be 0.5% of epidural analgesia for labor, rate increasing to 4% if top-ups are used for cesarean section anesthesia.^[3,4]^ Results of radiological studies considering wide local anatomical variations with ease of an accidental subdural puncture, lead us to think that its occurrence is probably underestimated.^[8]^

More often, it is considered as an unpredictable and unpreventable but also self-limited and benign event following epidural analgesia allowing the reuse of the catheter.^[1,2]^ It has also been related to inadvertent subdural injection of local anesthetics, which is a rare but potentially threatening complication of epidural analgesia, exposing to a potential risk of rupture of the arachnoid membrane.^[1,6]^

However, because of its multiple possible associated signs and even if it is a relatively well-described potential side effect of this anesthetic technique, its management remains unclear.

Some authors do not recommend to avoid the use of epidural catheter if Horner's syndrome occurred, but some others advise for neurologic evaluation of the patient with a history of Horner's syndrome after epidural catheterization before placing a catheter, in order not to ignore subdural complication.^[1,2]^

Recent echography developments allow anesthetists to insert the epineural catheter in difficult anatomical situations. However, no studies have been performed investigating the incidence of complications after catheter insertion, such as subdural puncture, by using this technique. It could be interesting, in the future, to quantify these events when ultrasound-guided catheter insertion is used.

## 4 Conclusion

To the best of our knowledge, only one clinical case of a repeated Horner’ syndrome following epidural analgesia after multiple attempts of catheterization has been reported in the literature. Particular local anatomy might explain our observations. Only a radiological study could have given us a clue. In our patient, epidural catheterization seems definitively contraindicated without imagery. The management of Horner's syndrome following epidural analgesia is not consensus-based and depends on neurologic evaluation. Diagnosis criteria for subdural blockade recognition should be developed in order to avoid the use of an unnoticed subdural catheter.

## Author contributions

**Supervision:** Jihad Mallat.

**Writing – original draft:** Caroline Turbelin, Jihad Mallat.
